# Preferences of dentists and endodontists, in Saudi Arabia, on management of necrotic pulp with acute apical abscess

**DOI:** 10.1186/s12903-018-0574-7

**Published:** 2018-06-19

**Authors:** Ahmad A. Madarati

**Affiliations:** 1 Restorative Dental Sciences Department, College of Dentistry, Taibah University, P.O Box 2898, Madina, 43353 Saudi Arabia; 2 Faculty of Dentistry, Aleppo University, Aleppo, Syria

**Keywords:** Acute, Apical, Abscess, Endodontics, Emergencies, Pus, Questionnaire, Necrotic, Survey

## Abstract

**Background:**

This study aimed at investigating dental clinicians’ preferences on management of necrotic pulp with acute apical abscess (NPAAA) cases.

**Methods:**

Following an ethical approval and two pilot studies, an electronic survey was emailed to 400 general dental practitioners (GDPs) and 56 endodontists. The email explained the study’s methods and assured that participants’ identities and information given would remain anonymous and confidential. A reminder email was sent after eight weeks. Responses were collected and data were analyzed using the Chi-square test at *p = 0.05*.

**Results:**

The majority of respondents (86.3%) would deal with NPAAA cases “*differently”* from vital-pulp ones (*p < 0.001*). More endodontists (40%) used two or three irrgants than GDPs (29.5%). Whilst the highest proportion of endodontists (29.7%) *rarely* prescribed antibiotics, the highest proportion of GDPs (26%) *generally did so* (*p < 0.001*). Whilst the highest proportion of GDPs (26.9%) *over-instrumented the largest canal* in the first visit, most endodontists (56.8%) performed *complete cleaning & shaping* (C&S) (*p < 0.001)*. In cases of non-stopped exudates, whilst the highest proportions of endodontists would either *let the patient wait till the exudates significantly reduce then continue their intended approach* (40.5%) or *insert ICMs and temporize the tooth* (40.5%), the highest proportion of GDPs (30.8%) would *insert only dry cotton pellet without temporizing the tooth (p = 0.002)*. Of those who would *leave the tooth open* if non-stopped exudates presents in the first visit, the majority (81.9%) would temporize the tooth if *little exudates* present after C&S *(p < 0.001)*.

**Conclusions:**

Clinicians, especially GDPs, opted to treat teeth involved in NPAAA differently from those with vital-pulp, such as: were using different ICMs and irrigants, C&S to different apical size preparation. GDPs should improve their practice by implementing multi-irrigants protocol while C&S, limit prescribing antibiotics, perform complete debridement of the root canal system and not to leave the tooth open between visits. Clinicians, especially GDPs, relied on their own experiences in managing NPAA cases which necessitates scientific-based guidelines.

**Electronic supplementary material:**

The online version of this article (10.1186/s12903-018-0574-7) contains supplementary material, which is available to authorized users.

## Background

The main objectives of root canal treatments are: a- to remove the infection from the root canal system (elimination of microbes), b- to perfectly seal the root canal system to prevent re-infection, and c- to promote healing of the periapical tissues. Accomplishment of these objectives, however, in some cases is challenging. Diagnosis and management of necrotic dental pulp cases that are associated with apical pathosis possess difficulties. Misdiagnosis or improper treatment procedures, due to lack of knowledge or insufficient clinical skills, may result in serious consequences for the patient. Clinicians must identify the etiology and mechanism of the periapical tissues’ pathosis and manage the case properly by providing the appropriate treatment measures. The role of bacteria and their toxic by-products in pathosis of the dental pulp and periradicular tissues is well established [1]. Therefore, after determining the corrected working length, complete canal debridement, with / without obturation, is the treatment of choice [2]. However, if the time is limited in the first visit, partial debridement at the estimated working length can be a practical and accepted therapeutic procedure [2]. It is suggested that the root canal system of the offended tooth is dried and then filled with calcium hydroxide and the coronal access cavity is sealed with a temporary filling [2]. While different inter-appointment medications can be recommended, there is a general belief that such a tooth should not be generally left open till next visit [2]. Moreover, in acute apical abscess, the use of systemic antibiotics has not been proved beneficial if the infection is localized [3] However, unfortunately general dental practitioners (GDPs) seem not to follow these recommended procedures. For example, in cases of pulp necrosis associated with periapical pathosis (especially acute apical abscess), leaving the tooth open has been a controversial, but disappointedly is a common practice, to some extent [4]. Walton and Keiser stated that placing a cotton pellet lightly dampened with an intracanal chemical medication in the pulp chamber before placing temporary filling is a useless procedure [2], though it is well adopted by GDPs. These are some of other procedures that GDPs may still follow without scientific evidence. Unfortunately, there has been lack of information regarding clinicians’ practices and preferences in this respect. Two previous surveys, conducted in the United States, showed how clinicians change their decision over period of time (5–7]. To this end, there is a need to explore management aspects and practitioners’ preferences and practices when dealing with cases of necrotic pulp associated with acute apical abscess (NPAAA). In particular, to date there are no reports on the real-life practice of GDPs and endodontists, neither in Saudi Arabia nor in any other country, when a case of NPAAA is encountered.

Therefore, the aim of this questionnaire study was to investigate the preferences, practices and experience of GDPs and endodontists in the Western Province, Saudi Arabia towards management of NPAAA cases. The survey mainly aimed to answer the following questions:

- ❏ Would be there any significant difference between clinicians (GDPs and endodontists) who used to leave the tooth with NPAAA open after the first visit and those clinicians who do not?
- ❏ Would be any significant difference between GDPs and endodontists regarding procedures adopted in the first visit of dealing with a tooth associated with NPAAA?

Therefore, the following two null hypotheses were tested:

- ❏ H0 (1): There would be no significant differences between clinicians (GDPs and endodontists) who would leave a tooth associated with NPAAA open in the first visit and those who wouldn’t.
- ❏ H0 (2): There would be no significant difference between GDPs and endodontists regarding some procedures followed in the first visit when dealing with a tooth associated with NPAAA open in the first visit.

## Methods

This Internet-based questionnaire study was ethically approved by the Research Ethics Committee at Taibah University College of Dentistry (Saudi Arabia) without the need for participants’ consent form. The study was accomplished in accordance to the World Medical Association’s Helsinki Declaration between March and August 2016. A first pilot survey study was conducted on the academic staff at College of Dentistry, Taibah University to ensure that the questions were easily understood without personal interpretation. An initial questionnaire comprised both close-ended and partially close-ended questions in two main categories:

- Demographics (three non-numbered questions)
- Pattern of practice and experience of management of cases associated with NPAAA (14 main questions)

A second pilot survey was performed on a group of GDPs and endodontists to facilitate the sample size calculation. The latter was performed taking into consideration the following factors:

- ❏ 90% power to detect the difference between groups’ proportions
- ❏ The expected (minimum desired) response rate (40–60%)
- ❏ The populations of the study target (number of GDPs and all endodontists in the Western Province, SA)
- ❏ Level of statistical significant difference (0.05)

It was decided that the survey would be sent to 400 GDPs and all endodontist registered in the Western Province, Saudi Arabia (56). The 400 GDPs were selected randomly using the systematic sampling method. The final questionnaire (Additional file 1) was constructed electronically using the Google Drive sheet. The electronic survey was emailed to the selected sample size (400 GDPs and 56 endodontists). The email explained the aims and methods of the study and assured that participants’ identities would remain anonymous and all information given would remain confidential and would be used for the purpose of the research only. A further email was sent to all candidates after eight weeks to remind non-respondents to participate in the survey. Responses were collected using the Google Drive Excel document. Data were entered into SPSS 19 for Windows software (SPSS Inc., Chicago, IL, USA) and they were analyzed using the Chi-square and Linear-by-Linear Association tests at *p = 0.05*. Statistical tests were performed to compare mainly among the proportions of each question responses and to compare between GDPs and endodontists regarding these responses.

## Results

### Classification of Respondents & Study’s response rate

Of the 456 who were approached, 234 responded to this study as follows: 189 (80.6%) were *GDPs*, 32 (13.7%) *endodontists*, 6 (2.6%) *postgraduate students or residents* in endodontic specialty programmes, and 7 (3%) *others*.

Eighteen respondents (16 GDPs and 2 others) never performed RCTs. Therefore, they were categorized ineligible respondents. Accordingly, the overall and non-endodontists (GDPs & otters) sample sizes were:

456–18 = 438 and 400–18 = 382, respectively, resulting in the following response rates:

- Overall response rate: 234 / 438 = 53.4%.
- Non-endodontists (GDPs, Endodontic Postgraduate Programmes students, and others) final response: 202 / 382: 52.9%
- Endodontists: 32 / 56 = 57.1%

This study involves mainly theoretical aspects of endodontics rather than attaining hand skills or mastering new techniques. Dealing with cases of NAPP is well established in the first year of the postgraduate studies programmes in Saudi Arabia. Therefore, participants who were enrolled in Endodontic Postgraduate Studies or Residency programmes, were categorized as endodontists. This also enabled better statistics for some variables.

### Participants’ experience & number of cases they perform per week

Overall, significantly the lowest proportion of respondents (8.8%) had *more than 20 years’ experience* (*p* < 0.001) (Table 1). The highest proportion of endodontists (44.4%) had *10.1 to 20 years’ experience* compared to 17.3% in GDPs group *(p = 0.005*).

**Table 1:** Participants’ experience, number of root canal treatments (RCTs) they perform per week and their approach for necrotic pulp associated with acute apical abscess (NPAAA) cases

Respondents’ Classification	Experience of Respondents (Years)					
	Up to 2	2.1 to 5	5.1 to 10	10.1 to 20	More than 20	Total
GDP	25.5%	25.4%	25.4%	17.3%	9.2%	100% (173)
Endodontists	0%	21.1%	26.3%	44.4%	7.9%	100% (38)
Others	0%	40%	20%	40%	0%	100% (5)
Total	18.1%	25%	25.5%	22.7%	8.8%	100% (216)
Respondents’ Classification	Number of RCT cases performed per week					
	1–5	6–10	11–15	More than 15	Total	
GDP	52%	28.3%	11.6%	8.1%	100% (173)	
Endodontists	10.5%	26.3%	18.4%	44.7%	100% (38)	
Other	80%	0%	20%	0%	100% (5)	
Total	45.4% (98)	27.3% (59)	13% (28)	14.4% (31)	100% (216)	
Respondents’ Classification	Management of permanent teeth with NPAAA					Total
	Treat the tooth		Extract the tooth	Refer to an endodontist		
GDP	91.2%		1.8%	7%		100% (173)
Endodontists	100%		0%	0%		100% (37)
Other	100%		0%	0%		100% (5)
Total	93.6%		1.3%	5.1%		100% (215)

There were significant differences between endodontists and GDPs regarding the number of RCTs performed per week (*p < 0.001)* (Table 1). Whilst the highest proportion of GDPs performed *1 to 5 cases* (52%), the highest proportion of endodontists (44.7%) performed *more than 15 cases***.**

Overall there were no significant differences between endodontists and GDPs (*p = 0.176)* regarding the decision on *how to deal with case associated with NPAAA* (Table 1). The vast majority of respondents (93.4%) *would treat the tooth (p < 0.001)* and only 8.8% of GDPs would either *refer to endodontic specialist* (7%) or *extract the tooth* (1.3%) *[p < 0.001].*

### Management’s differences between vital and NPAAA cases & main Treatment’s modalities adopted in NPAAA cases

Overall, the majority (86.3%) (Endodontists and GDPs) would deal with cases of NPAAA “*differently”* from that of vital ones (*p < 0.001*) (Table 2)*.*

**Table 2:** Management’s differences between vital and NPAAA cases & main treatment’s modalities adopted by Participants in NPAA cases

Would you deal with NPAA differently		Respondents’ Classification				Total	
		GDPs		Endodontists	Others		
Using of Rubber Dam		13.3%		9.7%	0%	11.9%	
Type of sealer		15.6%		6.5%	20%	13.5%	
Size of apical preparation		33.3%		32.3%	60%	34.1%	
Type of intra-canal medication		78.9%		16.1%	80%	63.5%	
Apical extension of preparation		17.8%		29%	20%	20.6%	
Methods for WL measurement		6.7%		3.2%	0%	5.6%	
Use of rotary instruments		8.9%		0%	20%	7.1%	
Technique of C&S		24.4%		22.6%	0%	23%	
Type of irrigants		62.2%		38.7%	100%	57.9%	
Obturation technique		17.8%		3.2%	0%	13.5%	
Overall Total		100% (90)		100% (31)	100% (5)	86.3% (126)	
Respondents’ Classification	Instrumentation techniques					Total	
	Conventional	Step Back		Crown Down			
GDP	21.2%	45.2%		57.7%		173 (100%)	
Endodontists	0%	8.6%		97.1%		38 (100%)	
Other	20%	60%		100%		5 (100%)	
Total	16%	36.8%		68.8%		216 (100%)	
	Irrigants used in NPAAA cases						Total
Respondents’ Classification	EDTA	NAOCL	CH	NAOCL + (EDTA or CH)^a^	NAOCL + (EDTA + CH)^a^	Other	
GDP	23.1%	92.3%	17.3%	29.5%	8.2%	3.8%	104 (100%)
Endodontists	21.6%	100%	10.8%	21.6%	10.8%	28%	37 (100%)
Other	40%	100%	20%	40%	20%	0%	5 (100%)
Total	23.3% (82.3%)	94.5%	15.8% (100%)	27.7%	9.1%	2.7%	141 (100%)
Respondents’ Classification	Patterns of Antibiotics Usage						Total
	Never	Always	Generally	Frequently	Sometimes	Rarely	
GDP	5.8%	21.2	26%	11.5%	24%	11.5%	100% (104)
Endodontists	13.5%	10.8%	10.8%	16.2%	18.9%	29.7%	100% (37)
Other	20%	20%	0%	0%	40%	20%	100% (5)
Total	8.2%	18.5%	21.2%	12.3%	23.3%	16.4%	100% (146)

The values in brackets represent proportion of those who used the assigned irrigant with sodium hypochlorite irrigant. ^a^Proportions were calculated out of sodium hypochlorite users

#### Size of apical preparation

34.1% would prepare root canals in cases with NPAAA to *different apical sizes* from those of vital pulps cases; without significant difference between endodontists and GDPs *(p = 0.913)* (Table 2)*.*

#### Type of intra-canal medications (ICMs)

Whist the majority of GDPs (78.9%) would use different ICMs when dealing with NPAAA cases compared to vital ones, only 16.1% of endodontists would do so *(p < 0.001)* (Table 2)*.*

#### Type of sealer

Only 13.5% reported the difference in the type of sealer used for RCT with significantly greater proportion of GDPs (15.6%) compared to that of endodontists (6.5%) *[p < 0.001]* (Table 2)*.*

#### Technique of cleaning & shaping (C&S)

23% would use different techniques for C&S of the root canal system in cases of NPAAA. Significantly, Crown *Down* was the most common technique used for C&S cases with NPAAA (68.8%) *[p < 0.001]*; with the vast majority of endodontists (97.1%) using it compared to significantly less GDPs (57.7%) *[p < 0.001]* (Table 2).

#### Type of Irrigants

Whilst the highest proportion of GDPs (62.2%) would use *different irrigants* during instrumentation of cases associated with NPAAA, only 38.7% would do so *(p = 0.023)* (Table 2)*.* Significantly, the vast majority of respondents (94.5%) used sodium hypochlorite irrigant *(p < 0.001)*. The proportion of endodontists who used two or three irrigants (40%) was significantly greater than that of GDPs (29.5%) *[p < 0.001]*.

#### Prescribing of antibiotics

Whilst the highest proportion of endodontists (29.7%) rarely prescribed antibiotics, only 11.5% of GDPs did so (*p = 0.004*). By contrast, the highest proportion of GDPs (26%) *generally prescribe antibiotics* (Table 2).

### First Visit’s management of NPAAA cases and its reasons

The most common management of NPAAA cases in the first visit were *complete C&S of the root canal system* and *over-instrumentation of the largest root canal* (*p < 0.001)*. Overall there were significant differences between endodontists and GDPs (*p < 0.001)* (Table 3).

**Table 3:** First visit’s management of NPAAA cases and the reasons participants report

Management of NPAAA in the first visit	Respondents’ classification			Total
	GDPs	Endodontists	Others	
Access Cavity	3.8%	5.4%	0%	4.1%
Over-instrumentation the largest canal	26.9%	8.1%	40%	22.6%
WL measurement	1.9%	0%	0%	2%
WL and C&S of the largest canal	7.7%	0%	0%	5.5%
Partial C&S at estimated WL	21.2%	0%	20%	15.8%
Partial C&S at Corrected WL	15.4%	8.1%	0%	13%
Complete C&S	19.2%	56.8%	40%	29.5%
Complete RCT	3.8%	21.6%	0%	8.2%
Overall Total	100% (104)	100% (37)	100% (5)	100% (146)
Reasons for first visit approach of NPAAA	Respondents’ classification			Total
	GDPs	Endodontists	Others	
Taught during undergraduate study	41.9%	27%	20%	37.4%
Lack of time	12.5%	2.7%	0%	9.6%
Learnt from own experience	56.2%	40.5%	40%	51.7%
Colleagues’ recommendation	16.5%	2.7%	20%	13.1%
Taught during postgraduation	0%	40%	60%	12.3%
Taught in a scientific meeting	1%	0%	0%	0.7%
Dental Literature	23.1%	32.4%	40%	26%
Total	100%	100%	100%	100%

Whilst the highest proportion of GDPs (26.9%) used to over-instrument the largest canal, the highest proportion of endodontists (56.8%) used to perform complete C&S (*p < 0.001)*. Also, while (21.2%) of GDPs used to *partially C&S the root canal system to the estimated working length*, none of endodontists (0%) did so (*p < 0.001)*. On the other hand, 21.6% of endodontists used *to perform complete RCTs*; with only 3.8% of GDPs were doing so (*p < 0.001)*. Overall, significantly the highest proportion (51.7%) reported their *own experience* as the reason for their first visit’s approaches to NPAAA cases followed by those who did it because *they were taught to do so during undergraduate training* (37.4%) *[p = 0.002]*. Forty percent of endodontists reported the *postgraduate studies* as the reason for doing so. By contrast, the highest proportion of GDPs (56.2%) reported their *own experience* as the reason for their first visit’s approaches.

### First Visit’s management of NPAAA cases with non-stopped exudates

Overall, there was no significant difference between the highest proportion of participants (41.4%) who used to *leave the tooth open* (without temporary restoration) and the lower proportion 36.3% who used to *place temporary restoration* if there was non-stopped excaudate after the first visit management (*p = 0.521*) (Table 4). However, significantly the lowest proportion (22.6%) would *let the patient wait till the exudates stop or significantly reduce then continue their intended approach [p = 0.018*]; with significantly more endodontists (40.5%) than GDPs (17.3%) *[p = 0.002]*. By contrast, the highest proportion of GDPs (30.8%) would *insert only dry cotton pellet without temporizing the tooth till next visit*. In addition, the proportion of endodontists who would insert ICMs and temporize the tooth (40.5%) was significantly greater than that of GDPs who would do the same (28.8%) *[p = 0.002]*. The proportion of endodontists who would temporize the tooth in the first visit (81.8%) was significantly greater than that of GDPs (44.2%).

**Table 4:** First visit’s management of NPAA cases with non-stopped exudates and the reasons participants report

First visit management of cases with non-stopped exudates		Respondents’ classification			Total	
		GDPs	Endodontists	Others		
Waite till the exudates stop or significantly reduce then continue the intended approach		17.3%	40.5%	0%	22.6%	
No temporary restoration	No intra-canal dressing	3.8%	5.4%	0%	4.1%	41.1%
	With dry cotton pellet	30.8%	5.4%	20%	24%	
	Insert intra-canal medication	11.5%	0%	0%	8.2%	
Place temporary restoration	Insert dry cotton pellet	7.7%	8.1%	20%	8.2%	36.3%
	Insert intra-canal medication	28.8%	40.5%	60%	32.9%	
Total		100% (104)	100% (37)	100% (5)	100% (146)	
Reasons for This Approach		Respondents’ classification			Total	
		GDPs	Endodontists	Others		
Taught during undergraduate study		22.3% (95.8)	0% (0)	20% (4.2)	16.6% (100)	
Lack of time		12.6% (100)	0% (0)	0% (0)	9% (100)	
Learnt from own experience		48.5% (78.1)	35.1% (20.3)	20% (1.6)	44.1% (100)	
Colleagues’ recommendation		6.8% (100)	0% (0)	0% (0)	4.8% (100)	
Taught during postgraduation		0% (0)	43.2% (88.9)	40% (11.1)	12.4% (100)	
Dental Literature		9.7% (52.6)	21.6% (42.1)	20 (5.3)	13.1% (100)	
Total		100%	100%	100%	100%	

While the most common reason for endodontists’ approaches (43.2%) was *postgraduate studies’ learning*, the most common reason for GDPs was *learning from their own experience (p < 0.001)* (Table 4).

### Management of NPAAA cases with little exudates after complete C&S and its reasons

The majority of respondents (80.8%) used to insert temporary restoration, with (69.2%) or without intracanal medications (11.6%), if there had been little exudates after the first visit management (Table 5). The second most common management (12.6%) was to *let the patient wait till the exudates stop or significantly reduce then continue their intended.* Overall, there were significant differences between GDPs and endodontists *(p < 0.010).* Whilst the second common approach by endodontists (29.7%) was *let the patient wait till the exudates stop or significantly reduce then continue their intended, insert dry cotton pellet only without temporizing the tooth till next visit* was the second most common management adopted by *GDPs* (15.4%). However, there was no significant difference between the proportion of endodontists who used to *insert temporary restoration with intracanal medications* (62.2%) and those of GDPs who were doing (70.2%) *[p < 0.097].*

**Table 5:** Management of NPAA cases with little exudates after complete cleaning and shaping (C&S) of the root canal system and the reasons participants report

First visit management of little exudates after complete C&S	Respondents’ classification			Total	
	GDPs	Endodontists	Others		
Waite till the exudates stop or significantly reduce then continue your intended approach	6.7%	29.7%	0%	12.3% (18)	
Leave tooth open without neither dressing nor temporization till next visit	1.9%	0%	0%	1.4%	6.8% (10)
Insert dry cotton pellet only without temporization till next visit	1.9%	5.4%	0%	2.7%	
Insert intra-canal medication without temporization till next visit	3.8%	0%	0%	2.7%	
Insert dry cotton pellet only and temporization till next visit	15.4%	2.7%	0%	11.6%	80.8% (118)
Insert intra-canal medication and temporization till next visit	70.2%	62.2%	100%	69.2%	
Total	100% (104)	100% (37)	100% (5)	100% (146)	

Overall, there were significant differences between endodontists and GDPs in reporting the reasons for their management of *little exudate after C&S* (*p > 0.05)*. The first and second most common reasons for GDPs were that they *were taught to do so during undergraduate study* and their *own experience* (52 and 46.1%, respectively). However, the most common reason for endodontists was that they *were taught to do so during postgraduate studies* (61.1%).

### Comparison between Management of First Visit Significant Exudates and Little Exudates after C&S

Of those who would *wait till the significant exudate stops then continue treatment in the first visit*, a significantly high proportion (54.5%, *p = 0.020)* would *temporize the tooth till next visit if little exudates still present after C&S of the root canal system in the first visit* (Table 6). Of those who would *leave the tooth open* if non-stopped exudates presents in the first visit, the majority (81.9%) would temporize the tooth if little exudates present after C&S *(p < 0.001)*. Of those who used to *temporize the tooth* after the first visit associated with non-stopped exudates, 5% *would wait till exudate stop then continue treatment* in case of little exudates presented in the first visit.

**Table 6:** Comparison between management of first visit significant exudates and little exudates after C&S

Management of First Visit Non-stopped Exudates	Management of First Visit Little Exudates after C&S			Total
	Wait till exudate stop then continue treatment	Leave tooth open till next visit	Temporize the tooth till next visit	
Wait till exudate stop then continue treatment	45.5%	0%	54.5%	100% (33)
Leave tooth without temporization till next visit	0%	18.9%	81.9%	100% (53)
Temporize the tooth till next visit	5%	0%	95%	100% (60)
Total	12.3%	6.8%	80.8%	100% (146)

## Discussion

There has been need to explore the real-life practice of GDPs and endodontists when dealing with cases of NPAAA and to identify the reasons behind their approaches. Generally, more endodontists had *more than ten years’ experience* than GDPs than GDPs. This might be explained by the fact that endodontists have to spend three to five years acquiring endodontic postgraduate training. As expected, there were significant differences between endodontists and GDPs regarding the *number of RCTs performed per week*; whilst most GDPs performed up to five cases per week, the highest proportion of endodontists performed more than 15 cases. While the full time of endodontists is devoted for endodontic treatment, GDPs usually perform other types of dental treatments. Another possible reason is that GDPs may refer complex cases or retreatment cases to endodontist [5]. The definition of complex cases from GDPs’ prospective should be established; because the vast majority of GDPs in the current study used to treat cases with NPAAA. It could be assumed that complex or difficult cases, from GDPs’ point of view, imply those cases with complex root canal anatomy or defective old root canal fillings. Nevertheless, these findings reflect participants’ intention to preserve teeth involved in NPAAA. This is especially true with the fact that 6.1% would refer the cases to endodontists and only (1.8%) would extract teeth involved with such cases. In addition, few years ago more GDPs in Saudi dental practice (5%) used to extract such cases [5]. However, dealing with NPAAA cases may be challenging and possess some difficulties. Accordingly, clinicians may deal with them differently when compared with vital-pulp cases. The majority of the current study’s respondents would do so. In the following we will try to discuss some of these different aspects, if any.

Almost 12% of respondents would use rubber dam (RD) isolation when dealing with NPAAA cases. This may explain the perception of these respondents towards the different pathological condition of NPAAA cases compared to vital-pulp cases. Different types of bacteria are associated or involved with different pathological endodontic conditions [6], hence these respondents were careful to prevent or reduce additional microbial invasion to the root canal system. RD isolation may also better control pus drainage. Nevertheless, it is well documented that RD is not commonly used by GDPs in many countries [7–10]. A recent study showed that only 21.6% of GDPs were using RD in Saudi Arabia [11]. While it is common, though disappointedly, for GDPs not to use RD, it is unaccepted that 9.7% of endodontists to use RD in NPAAA cases rather than vital-pulp cases. With the fact that RD isolation in endodontics is a standard of care [12].

Only 13.5% of participants (GDPs and endodontists) used to use different sealers when dealing with NPAAA cases. One assumption for such practice is that this group of clinicians’ demand better antimicrobial effects. The antimicrobial effects of zinc oxide & eugenol-based sealers is well established [13]. It has been thought that calcium hydroxide-based sealers would have better biological and antibacterial properties which could contribute to better perapical tissues healing and repair. However, clinical and experimental studies have not shown such superiority [14, 15]. The MTA-based sealer has been recently introduced and has shown promising results [16]. Nevertheless, drawing conclusions on the impact of different sealers on treatments’ long-term outcome should be based on clinical studies with sufficient follow-up periods. In addition, the current study did not ask the participants about the type of sealer they used in cases of NPAAA. This could be one limitation that necessitates further investigation.

There is a general belief that necrotic pulp cases, especially those associated with apical pathosis should be prepared to a larger apical size than vital-pulp cases. A previous clinical study found that enlarging the apical portion of root canals three sizes larger than the first file that bound at working length was sufficient [17]. Some authors have suggested creating a ‘larger’ apical preparation followed by a one-week dressing of calcium hydroxide [18–20]. Other authors have suggested enlarging the canal terminus to a pre-determined size beyond 35 or 40 [20–27]. However, it was suggested that larger tapers is more important than the final apical size; because a small taper size of 0.10 allowed minimum instrumentation of the apical part of the root canal systems [28]. One systematic review showed that canal enlargement reduces bioburden within the root canal system [29]. It revealed that in cases with necrotic pulps and periapical lesions, enlargement of the apical size would result in an increased healing outcome (clinically and radio-graphically) [29]. A very recent study revealed significant bacterial reduction when root canals of teeth involved with apical periodontitis were prepared to an S3 TFA file compared to S2 and S1 files [30]. The current study revealed that one third of participants were following this trend and they would prepare the root canals of NPAAA cases to different sizes of that in vital-pulp cases. Nevertheless, the influence of different apical enlargement on the treatment long-term outcome, especially in NPAAA cases, should be established systematically. This need was reflected on the current study; because two third of participants would not prepare the NPAAA cases to different apical size of that in vital-pulp cases. It is important to indicate that some studies proposed and investigated the interaction between the apical size preparation and other factors such as irrigation and obturation).

In addition to the antimicrobial and tissues- dissolving effects, irrigants are essentially used to remove and wash the debris out of the root canal system. Due to the extensive microbial invasion in NPAAA cases compared to those of vital-pulp ones, different irrigation protocols and stronger irrigants may be used [31]. Unlike endodontists, most GDPs in the current study used different irrigants when dealing with NPAAA cases. This again may reflect their perception to the pathological condition of the pulpal and periapical tissues in NPAAA cases when compared to vital-pulp cases. If this is the case, GDPs need to not underestimate the desired antimicrobial effects of irrigants in vital-pup cases as well. By contrast, the lowest proportion of endodontists used different irrigants. Endodontists are usually aware of the important of irrigation in elimination intracanal infection regardless of the pathological condition. There have been many solutions used as irrigants in endodontics; including sodium hypochlorite (NaOCl), chlorohexidine (CHX) and Ethylene diamine tetra acetic acid (EDTA). NaOCl has been the most commonly used irrigants [32]. The current study is not an exception and showed that the vast majority of clinicians were using it in NPAAA cases. It is an excellent antibacterial agent, capable of dissolving necrotic and vital tissues and the organic components of dentin and biofilms. Hence it has been the standard irrigant with which other and new irrigants are compared [33]. Chlorohexidine has been also known for its good antimicrobial effects, especially as adjunctive irrigant [34]. However, one limitation of both irrigants, is that their inferior ability in removing intracanal smear layer. Hence EDTA is strongly recommended for such a purpose [31]. An irrigation protocol that ensures elimination of microbs, dissolving pulp tissues and removing smear layer by combined use of selective irrigants in different sequences, is paramount. It is well established that such a protocol is key factor in better cleaning of the root canal system and contributes to better long-term treatment outcome [34]. Within this respect, the current study revealed the need to raise the awareness of GDPs for implementing multi-irrigants protocol as only one third of them use more than one irrigant.

The most common different practice of GDPs towards NPAAA cases from that in vital-pulp ones was using different type of ICMs; with only 16.1% of endodontists were doing so. This again, almost reflect GDPs perception of the significant microbial invasion and the essential need to eliminate intracranial infection. Also, it may mirror GDPs’ understanding of using different ICMs according to different pulpal and periapical pathological conditions and the associated symptoms. Nevertheless, the current results are consistent with those obtained in a very recent study conducted in Saudi Arabia which showed general trend among GDPs to use different ICMs according to different pulpal and periapical diseases [35]. These findings validate the current study and confirm that it was conducted systematically. The previous study revealed that CH was the most common ICM used by Saudi dental clinicians; GDPs and endodontists [35]. A previous study in the United States, also, found that CH was the most frequently used ICM all necrotic pulps cases [36]. The antimicrobial properties of CH dressing has been controversial [37], though it has been recommended for use in teeth with necrotic pulp tissue and bacterial contamination [2] because of its effectiveness in inhibiting microbial growth [38]. It probably has little benefit with vital pulps [2].

With NPAAA cases, extra caution and care should be exercised so the necrotic debris are not pushed apically, hence beyond the apex, during C&S to reduce post-treatment discomfort or pain. Crown-down instrumentation technique has been known as the least technique causing debris extrusion beyond the apex [39]. In addition, it enables removing the intracanal infection sequentially from the coronal portion down to the apical portion of the root canal system. The proportion of respondents, in the current study, who used this technique in cases with NPAAA was significantly greater than those who used Step-Back or Conventional ones. Unsurprisingly, the vast majority of endodontists used Crown-Down technique compared to lower proportion of GDPs. This indicates that GDPs need to update their knowledge and implement Crown-Down more as a technique of choice when dealing with NPAAA cases.

The results showed a clear trend among GDPs towards prescribing antibiotics when dealing with NPAAA cases. Whilst the highest proportion of endodontists (29.7%) rarely prescribed antibiotics, the highest proportion of GDPs (26%) generally prescribe. These findings are consistent with those obtained in a very recent systematic review [40]. However, the current results should be carefully interpreted because the current survey did not differentiate between presence and absence of swelling. This can be one limitation of the study which suggests further research to explore clinicians’ preferences on prescribing antibiotics for different endodontic diseases as well as to explore the most common antibiotics used in individual diseases. Systemic antibiotics are better prescribed for the diffuse, rapidly spreading or persistent infections with systemic signs and symptoms such as cellulitis or persistent swelling. The antibiotic therapy in such cases, is an adjunct to debridement of the root canal system [2]. A recent systematic review concluded that the use of systemic antibiotics is not necessary and not recommended if the the infection is localized [3]. Improper use of antibiotics is one main factor that contributes to the emerging oral bacteria resistance to commonly used antibiotics [41]. Improper use of antibiotics includes: use in cases with no infection, erroneous choice of the agent, dosage or duration of therapy, and excessive use in prophylaxis [42]. Nevertheless, the results indicate that clinicians, especially GDPs, should exercise additional care when prescribing antibiotics.

Management of NPAAA cases has been controversial, especially in terms of leaving the tooth open. Unfortunately, there is little evidence and reports on the best management and treatment modalities. An old survey in the United States revealed that most endodontists would do complete canal debridement shorter than the working length (WL) in cases no swelling present [43, 44]. By contrast, they would over-instrument the canals and leave the tooth open if there is swelling [43, 44]. Leaving the tooth open is no longer accepted nowadays and is rarely considered in very limited cases, especially for endodontists. A recent study in the United States revealed that most Endodontic Diplomats would do complete C&S regardless of the swelling; with up to 38% who left the tooth open in case of swelling [36]. It is well accepted among endodontists that full debridement of the root canal system, if time permits, in the first visit, is the preferred therapeutic procedure of NPAAA cases [2]. However, drainage should be considered if time is limited and does not allow complete C&S [2]. As expected, endodontists in the current study showed awareness of such good practice as most of them would C&S root canals to the full working length. By contrast, a lower proportion of GDPs would do so; with the highest proportion of them would over-instrument the largest canal only. Lack of time could be one main reason for this policy. Another possibility is that GDPs aim only at reducing the pressure and relief patients’ emergency situation. Interestingly, 21.6% of endodontists would perform complete RCT. The long-term treatment outcome and post-operative pain are the crucial debate in this respect. Reports indicated that there may be no difference in post-treatment pain if root canals are filled at the time of the emergency versus a later date [45] (Eleazer and Eleazer 1998). However, the long-term prognosis of such treatment is questionable [46, 47]. Nevertheless, a previous study showed no difference in treatment outcome between single-visit and two-visit treatments [48]. Overall, most clinician of the current study, especially GDPs, reported their *own experience* as the reason for their approaches to NPAAA cases in the first visit. This reflects the lack of scientific evidence that clinicians can rely on when dealing with such cases. This is especially true as most endodontist were equally relying on either their own experience or on what they were taught during postgraduate endodontic programmes.

Decision on NPAAA cases with significant exudates after first visit’s procedures is one of the most crucial decision-making skills, especially with the lack of strong scientific evidence [49]. This reflected clearly on GDPs options as the highest proportion reported their *own experience* as the main reason for their decision. This was confirmed, somehow, by endodontists group as it was the second most common reason. The highest proportion of GDPs (30.8%) would *insert dry cotton pellet only without temporizing the tooth till next visit*. Moreover, the trend within the GDPs group was to *leave the tooth open till next visit*. These findings are consistent with those obtained in a previous study which revealed that leaving the tooth open for drainage is still present in the United Kingdom’s dental practice [4]. With only 12% of GDPs, in the current study, reporting *lack of time* as a reason for their decision, it is clearly that they need to understanding that leaving the tooth open till next visit is inappropriate because it allows more microbial invasion to the root canal system and may cause more complications [49, 50]. Foreign objects may enter the root canal system or even to the periapical area [51]. With very little scientific evidence [49], the best action a clinician may take in case of significant exudates is that he/she steps away from the patient, or ask the patient to wait in the waiting area, for some time to allow the drainage to continue and hopefully resolve on the same treatment visit [2]. As expected, endodontic showed, to some extent, good practice as one of the highest proportions of them opted this good approach. Nevertheless, the trend of leaving the tooth open dramatically reduced in cases of little exudates after C&S. Of those who would leave the tooth open if non-stopped exudates presents in the first visit, the majority (81.9%) would temporize the tooth if little exudates present after C&S. It is clearly that the presence of the exudates impairs clinicians from taking the right decision. Clinicians need to understand that even with the presence of exudates, giving the tooth enough time in the first visit then C&S is usually the practice of choice that results in good drainage. More importantly, they need to improve their practice and do their best not to leave the tooth open regardless the tooth initial conditions.

Nevertheless, this study was conducted in Saudi dental practice, which can be considered as one limitation. Further studies in other countries with different dental practices environment, regulations and set-ups are paramount to conclude general recommendations.

## Conclusions

Within the limitations of the current study, the following can be concluded:

- Clinicians showed clear trend towards preserving teeth involved in NPAAA, though they, especially GDPs, opted to treat them differently from those with vital-pulp cases. The main differences were using different ICMs and different irrigants, C&S to different apical size preparation.
- GDPs need to improve their practicing in specific aspects when dealing with NPAAA cases such as implementing Crown-Down technique and multi-irrigants protocol while C&S, limit prescribing antibiotics, perform complete debridement of the root canal system and not to leave the tooth open between visits.
- Though endodontists showed overall good practice, they need to completely adhere to the obligatory use of rubber dam.
- There is urgent need for clear guidelines that are based on scientific evidence because clinicians, especially GDPs, relied on their own experiences in managing NPAA cases.

## Additional file

Additional file 1: The survey form of Preferences of Dentists and endodontists, on Management of Necrotic Pulp with Acute Apical Abscess. (DOCX 39 kb)

## Abbreviations

C&S: cleaning and shaping
CH: calcium hydroxide
CHX: chlorohexidine
EDTA: ethylene diamine tetra acetic acid
GDPs: general dental practitioners
NPAAA: necrotic pulp with acute apical abscess
