# Supplementary material for: Preferences of dentists and endodontists, in Saudi Arabia, on management of necrotic pulp with acute apical abscess
Source: BMC Oral Health. 2018 Jun 19;18:110. doi: 10.1186/s12903-018-0574-7 (PMC6009056; doi:10.1186/s12903-018-0574-7)
Supplement: Supplementary file 1 — The survey form of Preferences of Dentists and endodontists, on Management of Necrotic Pulp with Acute Apical Abscess. (DOCX 39 kb) [file 12903_2018_574_MOESM1_ESM.docx]

***General information:***

**How long (years), roughly, have you been practicing dentistry?**

- Up to 2
- 2.1- 5
- 5.1-10
- 10.1-20
- More than 20

**Are you? (Tick as you want)**

- General dentist
- Endodontist
- Resident or postgraduate student in endodontics
- Specialist, residents or postgraduate student in another speciality

1. **On average, how many Root Canal Treatments (for permanent teeth) do you perform PER WEEK?**

- Never do root canal treatment (This is the end of the survey for you, thank you for participation)
- 1 to 5 cases
- 6 to 10 cases
- 11 to 15 cases
- More than 15 cases

1. **What do you USUALLY do with such cases? (Only one answer)**

- Treat the tooth (Please go to Q3)
- Extract the tooth (This is the end of the survey for you, thank you for participation)
- Refer the patient to an endodontist (This is the end of the survey for you, thank you for participation)

1. **Would you deal with this case differently from that of Vital Pulp regarding any of the followings? (Tick as you want)**

- Administration of Anaesthesia
- Using of rubber
- Types of sealer
- Size of apical preparation
- Using of rotary systems
- Technique for obturation
- Using of inter-appointments medication
- Type of inter-appointment medication
- Method(s) of measuring working length
- Technique of canal instrumentation
- Apical extension of preparation and obturation (there is no apex resorption)
- Types of irrigants, if any
- Removal of smear layer
- No differences

1. **In the first visit, what do you USUALLY perform? (Only one answer)**

- **Only** Access Cavity Preparation
- Instrumentation of the **largest canal**, beyond the apex, to allow drainage of pus, if any
- Measurement of the Working Length (WL)
- Measurement of WL and instrumentation of the **largest canal** for drainage
- **Partial** cleaning and shaping at the **Corrected** WL
- **Complete** cleaning and shaping at the **Corrected** WL
- Complete root canal treatment (RCT), if the canals **are dry** after instrumentation

**You do this (answer for Q 4) because (Tick as you want):**

- You were taught to do so while being undergraduate
- You learnt from your experience
- Your colleges' recommendation
- You were taught to do so while being postgraduate
- Lack of Time
- You were taught to do so in a scientific meeting

1. **If the purulence is persistent and obstructs what you intended to do, what would you USUALLY do? (Only one answer)**

- Let the patient sit for a time in the clinic till the purulence stops or significantly reduces, then continue doing what you intended to do.
- Leave the tooth opened **withou**t neither cotton pellet nor temporary restoration till next visit
- Place **dry cotton pellet** and leave the tooth open, **without** temporary filling, till next visit.
- Place **dry cotton pellet** and **temporize the tooth** till next visit.
- Place inter-appointment medication and leave the tooth **without** temporary filling till next visit.
- Place inter-appointment medication and **temporize the tooth** till next visit

**You do this because (Tick as you want):**

- You were taught to do so while being undergraduate
- You learnt from your experience
- Lack of Time
- Your colleges' recommendation
- You were taught to do so while being postgraduate
- You were taught to do so in a scientific meeting

**What is the inter-appointment medication(s) do you usually use in your answer to Q5?**

- Anaesthesia solution
- Sodium hypochlorite
- Formocresol
- Eugenol
- Ledermix
- Iodoform OR Iodoform-based
- Antibiotics mixture
- Non-setting Calcium hydroxide OR calcium hydroxide-based
- Camphorated mono chlorophenol (CMCP)
- Combination, please
- MTAD

1. **If, after complete root-canal cleaning and shaping, there is SLIGHT (little) intra-canal exudates, what would you USUALLY do?**

- Let the patient sit for a time in the clinic till the purulence stops, then continue doing what you intended to do.
- Leave the tooth opened **withou**t neither cotton pellet nor temporary filling till next visit
- Place **dry cotton pellet** and leave the tooth **without** temporary filling till next visit.
- Place inter-appointment medication and leave the tooth **without** temporary filling till next visit.
- Place **dry cotton pellet** and **temporize the tooth** till next visit.
- Place inter-appointment medication and **temporize the** tooth till next visit

1. **What is the technique do you usually use for root canal cleaning and shaping in such cases? (Tick as you want)**

- Conventional (standardaized
- Step Back
- Crown Down
- Others; please specify

1. **What is the root canal irrigant(s) do you use in such cases? (Tick as you want)**

- Never Use Irrigants
- Saline
- EDTA
- Sodium Hypochlorite
- MTAD
- Local Aaesthesua solution
- Chloramine
- Sterile water
- Hydrogen peroxide
- Chlorhexidine
- Combination of irrigants, please specify:

1. **How often do you prescribe antibiotics while dealing with such a case?**

- Always (100%)
- Generally (90-99%)
- Frequently (50-89%)
- Sometimes (10-49%)
- Rarely (1-9%)
- Never (0%)

***This is the end of the questionnaire, Thank you for your participation***
